# A comparative study of efficacy of propofol auto-co-induction versus midazolam propofol co-induction using the priming principle

**DOI:** 10.4103/0019-5049.72647

**Published:** 2010

**Authors:** Roopam Kataria, Ajay Singhal, Sukirti Prakash, Ishwar Singh

**Affiliations:** Department of Anaesthesiology and Intensive Care, Jaipur Golden Hospital, New Delhi, India

**Keywords:** Priming principle, propofol, auto-co-induction, bispectral index

## Abstract

Application of priming principle is well documented in relation to the use of muscle relaxants. The aim of the present study was to evaluate the efficacy of priming technique in relation to induction agents. Clinical efficacy in terms of dose reduction and alteration in peri-intubation haemodynamics was compared in propofol auto-co-induction and midazolam propofol co-induction groups along with a control group. The study was carried out in 90 patients scheduled for upper abdominal surgery, who were randomly divided into three equal groups. Group I received 0.5 mg/kg propofol IV (20% of the pre-calculated induction dose), group II received 0.05 mg/kg IV midazolam and group III received 3 ml of normal saline. This was followed by IV induction with propofol 2 minutes later in all the three groups at a predetermined rate till the bispectral index value of 45 was attained. The results showed a significant decrease in induction dose requirement in both the groups but haemodynamic stability during induction and intubation was more in propofol auto-co-induction group.

## INTRODUCTION

“Auto-co-induction”[1,2] is a technique of giving a pre-calculated dose of induction agent prior to giving the full dose of same induction agent; this technique is also known as “the priming technique”.[3] Application of priming principle is well documented in relation to the use of muscle relaxants. The priming technique involves giving a small sub-paralysing dose of the non-depolariser[4] (20% of the ED_95_ or about 10% of the intubating dose), 2–4 minutes prior to administering the second large dose for tracheal intubation.

“Co-induction”[5–7] is defined as the concurrent administration of two or more drugs that facilitate induction of anaesthesia documenting *synergism*.[8,9]

However, there is a paucity of studies[3] documenting the application of priming principle in induction agents. This technique, in relation to induction agents, aims at utilising the sedative, anxiolytic and amnesic properties at sub-hypnotic dosage of induction agent when given a few minutes prior to induction. This study was also done to evaluate whether the priming technique reduces the effective dose of induction agent and favourably influences the peri-intubation haemodynamics. Propofol and midazolam is a commonly used combination for induction and it shows synergistic interaction for hypnosis and reflex sympathetic suppression.[10–12]

## METHODS

The present study was conducted in our department after obtaining the approval of Institutional Ethical Committee. Ninety patients of age between 18 and 50 years, American Society of Anesthesiologist (ASA) Grade I and II, from both sexes having no history of adverse anaesthetic reaction, were randomly allocated into three equal groups: group I (propofol), group II (midazolam) and group III (normal saline), consisting of 30 patients in each.

In the operating room, routine monitoring, i.e., non-invasive blood pressure (NIBP), pulse oximetry and continuous surface ECG, was used. Along with these, Bispectral Index (BIS) monitor BIS xp model no.A-2000 (Aspect Medical Systems Inc., Norwood, USA) was used. Fronto-temporal (BIS Quatro) surface electrodes were placed on the patient’s forehead, after skin preparation. The impedance of electrodes was checked and smoothing rate was set at 15 seconds. Pre-operative baseline values of heart rate (HR) and blood pressure (BP) (an average of two consecutive readings) were taken 5 minutes apart before the induction of anaesthesia. Baseline BIS value were also recorded. An intravenous line appropriate for the surgical procedure was secured in the left upper limb.

Patients in the three groups (I, II, III) received the priming agent 0.5 mg/kg IV propofol, 0.05 mg/kg IV midazolam and 3 ml of normal saline, respectively, followed by IV induction with propofol 2 minutes later in all the three groups until the BIS value of 45 was achieved. The speed of injecting IV induction dose of propofol in all cases was at the rate of 30 mg/10 seconds. Any complication during this period, i.e., apnoea, vomiting, laryngospasm, involuntary movements, coughing, was noted.

Subsequent relaxation and intubation were accomplished with Inj. Rocuronium 1 mg/kg IV and anaesthesia was maintained on O_2_/N_2_O (35%, 65%); inhalational agent, i.e., Isoflurane and injection Vecuronium (0.02 mg/kg). No stimuli were applied during the 5-minute post-intubation period.

The following parameters were recorded.

- Total dose of propofol required in achieving targeted BIS value.
- SpO_2_, BIS value, HR and NIBP [(systolic blood pressure (SBP) and diastolic blood pressure (DBP)] were measured just before induction, immediately after induction, immediately after intubation, and 5 minutes after intubation.
- Post-operative recall phenomenon was also inquired for.

## RESULTS

The sample size of this study was calculated based on assuming a power of 80% and α (alpha) value of 0.05 as significant using epi info software (version 6.0). All data were reported as mean value ±2 SD.

The data were analysed statistically using the SPSS statistical package (version 10.0). Comparison between the groups for the induction dose and haemodynamic parameters was done using analysis of variance (ANOVA) with Tukey’s *post-hoc* test. A *P* value of <0.05 was considered to be significant and *P*<0.001 was considered to be highly significant.

The demographic data were comparable for age, weight, gender and ASA grading among the three groups, as shown in Table 1.

**Table 1:** Sociodemographic details

Groups	Mean age (years)	Sex distribution M:F	ASA grade I:II	Mean body wt. (kg)
Propofol I	32.3 (9.97)	15:15	23:7	59.7 (17.6)
Midazolam II	34.27 (7.89)	10:20	21:9	60.1 (10.54)
Normal saline III	31.33 (8.50)	14:16	19:11	63.10 (11.16)
*P* value	0.407	0.387	0.530	0.594

Mean values given along with 2SD given in brackets, Statistically not significant, ASA: American Society of Anesthesiologist

A statistically significant difference (*P*<0.001) was observed in propofol induction dose requirement in groups I and II compared to the control group. Mean induction dose requirement was found to be 45.37% lesser in midazolam co-induction group and 31.88% lesser in propofol auto-co-induction group as compared to the control group [Table 2].

**Table 2:** Mean Propofol induction dose used in the three groups

	Groups			*P* (Tukey’s *post-hoc* test)
	Propofol I	Midazolam II	Normal saline III	
Propofol induction dose (mg)	75.7 (19.78)	60.7 (15.74)	111.13* (31.65)	<0.001

Mean values given along with 2SD given in brackets,

* Statistically significant from other two groups

A statistically significant (*P*<0.001) difference was observed in post-priming BIS values among all the three groups. Maximum fall at post-induction interval was found in the propofol group. No variability was observed in BIS values at post-induction, post-intubation and 5 minutes post-intubation for both the study groups, i.e., propofol auto-co-induction and midazolam co-induction groups [Table 3].

**Table 3:** BIS values among three groups

BIS values	Groups			*P* (Tukey’s *post-hoc* test)
	Propofol I	Midazolam II	Normal saline III	
Baseline	96.40	97.10	96.56	0.133
Post-priming	75.60*	82.40*	94.40*	<0.001
Post-induction	41.36	41.03	43.20	0.055

* Statistically significant between these two groups,

BIS: Bispectral Index

No variability was observed in mean SpO_2_ value at any interval during the study in all the three groups.

A statistically significant (*P*<0.001) fall in HR was observed in propofol auto-co-induction group at the post-priming interval. Post-intubation rise in the HR was observed in all the three groups but the least rise was found in the propofol group (group I).

Mean SBP was observed to be maintained at induction in the control group and a slight fall was observed in other two groups. Maximum rise in SBP after intubation (20.19%) from pre-induction value was observed in the midazolam co-induction group.

Mean DBP was observed to be maintained in control group at induction (with a slight fall observed in other two groups). Maximum fall in DBP (17.99%) from pre-induction value at post-induction interval was observed in propofol auto-co-induction group. Maximum rise in DBP (16.80%) from baseline value at post-intubation interval was observed in midazolam auto-co-induction group [Table 4].

**Table 4:** HR, SBP and DBP values for all the three groups

Variables	HR			SBP			DBP		
Time intervals	Group I	Group II	Group III	Group I	Group II	Group III	Group I	Group II	Group III
Baseline	82.83	82.80	84.30	130.80	124.16	129.53	79.3	79.96	82.06
Post-priming	80.16*	84.06	90.40*	119.43	117.06	130.00**	71.96	74.7	85.16**
Post-induction	80.23	83.70	91.73**	109.86	115.26	116.46	65.03**	74.63	73.20
Post-intubation	99.43*	105.80	110.13*	136.26	149.23**	134.80	85.96	104.40**	90.96
5 minutes post-intubation	98.73	106.16	104.93	131.83	130.93	126.93	82.13	84.23	82.53

* Statistically significant between these two groups,

** Statistically significant from other two groups

HR: Heart rate, SBP: Systolic blood pressure, DBP: Diastolic blood presssure

## DISCUSSION

The present study was conducted to evaluate the clinical efficacy of propofol auto co-induction as compared to midazolam propofol co-induction, in terms of reduction in the induction dose of propofol and better haemodynamic stability in peri-intubation period.

In group I, after priming with propofol, mean induction dose requirement of propofol [Table 2] was 75.70 mg as compared to the mean induction dose of 111 mg in the control group. We observed a 31.88% reduction in induction dose of propofol by applying auto-co-induction. Previous studies have[1,3] supported the above observation that propofol predosing significantly reduces its induction dose. Anil Kumar and colleagues[2] have found 27.48% reduction in induction dose requirement of propofol after propofol auto-co-induction. The amnesic and sedative action of propofol at sub-hypnotic doses may facilitate the induction of anaesthesia at a lower induction dose of propofol.[13] In group II, after priming with midazolam, mean induction dose of propofol [Table 2] was 60.70 mg as compared to the mean induction dose of 111 mg in the control group. There was 45.37% reduction in the induction dose of propofol with midazolam co-induction. Earlier studies[14,15] also support the reduction in the induction dose of propofol after midazolam pre-treatment.

In the present study a predetermined BIS value (i.e., BIS 45) was taken as an endpoint of induction.[16,17] The maximum reduction in BIS [Table 3] at post-priming interval was found in propofol auto-co-induction group; but contrary to that, reduction in the induction dose requirement of propofol was maximal in midazolam group.

There was a significantly lesser fall in both SBP and DBP in propofol group at the post-induction interval. Propofol reduces BP by reducing vascular smooth muscle tone and total peripheral resistance and also by decreasing sympathetic activity. The lesser fall in propofol group was probably because of reduction in total induction dose of propofol after its auto-co-induction. The finding of less post-induction hypotension with significant dose reduction in propofol auto-co-induction group in the studies carried out by Djaiani *et al*.[1] also support the above observation. The rise in HR secondary to intubation [Table 4] was observed in all the study groups but it was significantly lesser in propofol auto-co-induction group. The rise in SBP and DBP [Table 4] immediately after intubation and 5 minutes post-intubation was significantly higher in midazolam co-induction group. The rise in SBP and DBP in propofol auto-co-induction group was comparable to the control group where much higher induction dose of propofol was used. Although propofol pre-treatment does not completely attenuate reflex sympathetic stimulation secondary to intubation, it is definitely more advantageous than the other two groups.

These observations point that although midazolam co-induction significantly reduces the induction dose of propofol, it does not provide haemodynamic stability in peri-intubation period. Similar results were also obtained by Cressy *et al*.[14] where significant dose reduction in propofol was found in midazolam pre-treatment group but there were no demonstrable benefits in terms of cardiovascular stability.

## CONCLUSIONS

The present study compared the efficacy of propofol auto-co-induction versus midazolam propofol co-induction. The following conclusions and inferences can be drawn from this study:

1. A significant fall in the induction dose requirement of propofol is found in both the study groups.
2. The priming in relation to propofol provides haemodynamic stability both at post-induction interval and secondary to intubation.
3. The priming in relation to propofol also appears to be cost effective by significantly reducing the total dose of propofol required.
4. However, more studies with larger samples are required before considering these observations as generalised.
